# Mirna Expression Profiles Identify Drivers in Colorectal and Pancreatic Cancers

**DOI:** 10.1371/journal.pone.0033663

**Published:** 2012-03-30

**Authors:** Ada Piepoli, Francesca Tavano, Massimiliano Copetti, Tommaso Mazza, Orazio Palumbo, Anna Panza, Francesco Fabio di Mola, Valerio Pazienza, Gianluigi Mazzoccoli, Giuseppe Biscaglia, Annamaria Gentile, Nicola Mastrodonato, Massimo Carella, Fabio Pellegrini, Pierluigi di Sebastiano, Angelo Andriulli

**Affiliations:** 1 Department and Laboratory of Gastroenterology, IRCCS “Casa Sollievo della Sofferenza”, Research Hospital, San Giovanni Rotondo, Italy; 2 Department of Surgery, IRCCS “Casa Sollievo della Sofferenza”, Research Hospital, San Giovanni Rotondo, Italy; 3 Unit of Biostatistics, IRCCS “Casa Sollievo della Sofferenza”, Research Hospital, San Giovanni Rotondo, Italy; 4 Unit of Bioinformatics, IRCCS “Casa Sollievo della Sofferenza”, Research Hospital, San Giovanni Rotondo, Italy; 5 Medical Genetics Unit, IRCCS “Casa Sollievo della Sofferenza”, Research Hospital, San Giovanni Rotondo, Italy; 6 Department of Internal Medicine and Chronobiology Unit, IRCCS “Casa Sollievo della Sofferenza”, Research Hospital, San Giovanni Rotondo, Italy; 7 Unit of Biostatistics, DCPE Consorzio Mario Negri Sud, Santa Maria Imbaro, Italy; University of Barcelona, Spain

## Abstract

**Background and Aim:**

Altered expression of microRNAs (miRNAs) hallmarks many cancer types. The study of the associations of miRNA expression profile and cancer phenotype could help identify the links between deregulation of miRNA expression and oncogenic pathways.

**Methods:**

Expression profiling of 866 human miRNAs in 19 colorectal and 17 pancreatic cancers and in matched adjacent normal tissues was investigated. Classical paired t-test and random forest analyses were applied to identify miRNAs associated with tissue-specific tumors. Network analysis based on a computational approach to mine associations between cancer types and miRNAs was performed.

**Results:**

The merge between the two statistical methods used to intersect the miRNAs differentially expressed in colon and pancreatic cancers allowed the identification of cancer-specific miRNA alterations. By miRNA-network analysis, tissue-specific patterns of miRNA deregulation were traced: the driving miRNAs were *miR-195, miR-1280, miR-140-3p* and *miR-1246* in colorectal tumors, and *miR-103, miR-23a* and *miR-15b* in pancreatic cancers.

**Conclusion:**

MiRNA expression profiles may identify cancer-specific signatures and potentially useful biomarkers for the diagnosis of tissue specific cancers. miRNA-network analysis help identify altered miRNA regulatory networks that could play a role in tumor pathogenesis.

## Introduction

MicroRNAs (miRNAs) are small non-protein coding RNA molecules that regulate gene expression mainly at the level of protein synthesis [1]. They represent an evolutionary highly conserved system that controls crucial cellular processes, such as development, differentiation, proliferation, apoptosis, and metabolism [2]. Aberrant expression of miRNAs can arise from deletion, mutation, and methylation of miRNA-encoding genes, many of which located at genomic fragile sites or regions frequently deleted or amplified in cancer [3]. Based on these premises, they have been proven to interact with potential oncogenes or tumor suppressors [4]. Many miRNAs are expressed in a tissue-specific manner, with profiles differentially expressed in either normal and neoplastic tissues, and in tumors with distinct biological properties. In addition, some evidences indicate miRNA profiles to allow reliable identification of the cell-of-origin of tumors [5].

Classically, differential expression of miRNAs has been evaluated either as single contribution or as predictive signature [6], [7]. However, much of scientific effort is spent currently to elucidate their functions: most miRNAs control at least one mRNA, and most mRNAs are controlled by more than one miRNA [8]. The complexity behind this sophisticated mechanism can be partly alleviated by the knowledge of the conservation levels of each miRNA. Indeed, high conservation across wide phylogenetic distances helps restricting the range of functions that a miRNA can play to regulate the control of species development and physiology.

In this study, we investigated expression patterns of miRNAs in colorectal (CRC) and pancreatic cancer (PC) with the intent to identify miRNA regulatory networks likely involved in oncogenic pathways by evaluating cancer-specific signatures through the analysis of the relationship between miRNA expression profile and cancer lineages.

## Materials and Methods

### Samples Selection and RNA Extraction

Primary tumor and neighbouring non-tumorous tissues were obtained from two training cohorts of 19 CRC patients and 17 PC patients, and from two validation cohorts of 14 CRC and 21 PC patients.. The study was performed with the approval of the Scientific and Ethic committees of the IRCCS “Casa Sollievo della Sofferenza” Institute, San Giovanni Rotondo, FG (Italy). The patients gave informed written consent according to the Italian law on privacy (providing for the protection of personal data), so individuals can not be identified from data or images included in this publication, and approved by the Scientific and Ethic committees of the IRCCS “Casa Sollievo della Sofferenza” Institute. All clinical investigations have been conducted according to the principles expressed in the Declaration of Helsinki.

Patient clinical data, tumor location, and staging are shown in Table S1. Tissue samples were flash frozen in liquid nitrogen and stored at −80°C until nucleic acids extraction. About 150–200 mg fresh frozen tissues were used to isolate total RNA by phenol extraction (TRIzol Reagent, Invitrogen Corporation, Carlsbad, CA, USA). RNA concentration and purity were controlled by Nano Drop Specthophotometer. The Agilent 2100 Bioanalyzer was used to measure the quantity, integrity and purity of small RNAs and total RNA, and only non degraded RNA characterized by an RNA integrity number >7 with no DNA contamination signs was processed.

### MiRNA Microarrays

MiRNA expression profile was determined by using GeneChip® miRNA Array (www.affymetrix.com). This array contains 46,228 probes comprising 7,815 probe sets, including controls, and covers 71 organisms such as human, mouse, rat and dog. Content is derived from the Sanger miRBase miRNA database v.11 (April 15, 2008, http://microrna.sanger.ac.uk). Probe sets targeting human snoRNAs and scaRNAs are derived from the snoRNABase (www.snorna.biotoul.fr/coordinates.php) and the Ensembl (www.ensembl.org/biomart/martview) archives, Briefly, 1.5 µg of total RNA was labeled using the 3DNA Array Detection FlashTag™ RNA Labeling Kit (http://www.genisphere.com), according to manufacturer's recommendations. First, poly (A) tailing was carried out at 37°C for 15 min in a volume of 15 ml reaction mix, which contained 1X Reaction Buffer, 1.5 ml MgCl2 [25 mM], 1 µl ATP Mix diluted 1∶500 and 1 µl PAP enzyme. Second, Flash Tag Ligation was performed at room temperature for 30 min by adding 4 µl of 5X Flash Tag Ligation Mix Biotin and 2 µl T4 DNA Ligase into the 15 µl of reaction mix. To stop the reaction, 2.5 µl of Stop Solution was added. Samples were hybridized, washed and scanned with an Affymetrix Scanner.

All microarray data are MIAME compliant and the raw data has been deposited in ArrayExpress (accession number E-MTAB-752 for colon cancer miRNA expression profiles and E-MTAB-753 for pancreatic cancer miRNA expression profiles).

### Quantitative estimation of miRNA by reverse transcription Real-Time PCR assay

Quantitative real-time polymerase chain reaction (qPCR) of miRNAs was performed using TaqMan MicroRNA Assay (Applied Biosystems, Foster City, CA, USA) with ABI-PRISM 7700 Sequence Detection System. A two-step protocol requires reverse transcription with a miRNA-specific primer, followed by a real-time PCR with TaqMan probes. The assays target only mature miRNAs, not their precursors. In brief, reverse transcriptase reactions contained 10 ng of RNA samples, 50 nM stemloop RT primer, 10X RT buffer, 0.25 mM each of dNTPs, 3.33 U/µl MultiScribe RT and 0.25 U/µl RNase inhibitor (all purchased from cDNA Archive kit of Applied Biosystems) were performed in final volume of 15 µl and incubated in termal cycler for 30 min at 16°C, 30 min at 42°C, 5 min at 85°C and then held at 4°C. The 20 µl PCR reaction mixture included 1.3 µl RT product (1∶15 diluition), 10 µl of TaqMan 2X Universal PCR Master Mix (NoUmpErase UNG) and 1 µl of TaqMan 20X MicroRNA Assay (Applied Biosystems, Foster City, CA, USA). Reactions were incubated in a 96-well optical plate at 95°C for 10 min, followed by 40 cycles of 95°C for 15 sec and 60°C for 10 min. All assays were performed in triplicate.

### Statistical Analysis

Patients' baseline characteristics were reported as mean ± standard deviation (SD) or frequencies and percentages for continuous and categorical variables, respectively.

For each of the two training cohorts, microRNA chip data were normalized using Robust Multi-array Average (RMA) algorithm [9]. In order to identify differentially expressed miRNAs between paired normal and tumor tissues, two approaches were followed. Firstly, a classical paired t-test controlling for false discovery rate (fdr) allowed ranking miRNAs according to their p-values. Secondly, random forest (RF) analysis [10] was applied to detect miRNAs with the best capability in discriminating tumor from paired normal tissues. A RF is a classifier consisting of an ensemble of tree-structured classifiers. According to this technique, 100,000 trees were built to classify tissues. The learning set used to grow each tree was a .632+ bootstrap resample of the observations. Trees were allowed to grow to their full size without pruning. The best split at each node was selected from a random subset of miRNAs. The left-out observations (i.e., “out of bag” observations) were then predicted to obtain the classification error rate of the considered tree. Predictive ability of the classifier was assessed aggregating the single tree error rates. Furthermore, the random forest framework allowed us to estimate the importance of a variable by looking at how much the classification error increases when “out of bag” data for that variable are permuted while all others are left unchanged. The importance metric used was the Mean Decrease in Accuracy (MDA). The MDA is constructed by permuting the values of each variable of the test set, recording the prediction and comparing it with the un-permuted test set prediction of the variable. Therefore, it is the increase in the percentage of times a test set is misclassified when the variable is permuted. We followed Strobl et al. [11] to avoid possible bias in variable selection: individual classification trees were built using subsampling without replacement and adopting a conditional permutation scheme [12]. We obtained a miRNAs ranking in accordance to the variable importance measure. Finally, the two miRNA rankings, one from the classical analysis and one from RF analysis, were merged to obtain a list of differentially expressed miRNAs. Correlations between miRNA expression were estimated using Spearman coefficient. A p value<0.05 was considered for statistical significance. All analyses were performed using SAS Release 9.1 (SAS Institute, Cary, NC, USA) and R for random forest analyses (*randomForest* package).

Quantitative real-time PCR (qPCR) assay was used on external validation cohorts to confirm microRNA array results, which were assessed using the Comparative Threshold cycle (CT) method and default threshold settings. Relative expressions of miRNA were computed by 2^−ΔΔCt^ formula [13] using U6 small nuclear RNA (RNU6B) as normalization control. Since the 2^−ΔΔCt^ transformed values were not normally distributed, comparisons were made using the non-parametric Wilcoxon signed rank test in order to assess the statistical significance of the up or down regulation. For the qPCR validation analyses, we considered a sample size of 14 subjects to provide a 90% power (with a two-sided alpha = 0.05) to detect an up-regulation of at least 2 or a down-regulation of at most 0.5 of each miRNA's expression in terms of 2^−ΔΔCt^ using the non-parametric Wilcoxon signed rank test and 1 as the null hypothesis.

### Network analysis

For each of the two training cohorts an undirected and weighted network was built, where nodes and edges represent miRNAs and their correlations (when significant), respectively. Thus, edges are not oriented since correlation is symmetric, and weighted with the Spearman coefficient. The thickness of edges mirrors the magnitude of the weights. In order to dig critical nodes out of both CRC and PC networks, we borrowed some methods from the social sciences, and evaluated the following *centrality* measures: *degree*, *betweenness* and *clustering coefficient* [14], [15] for each network. Finally, miRNAs have been ranked accordingly.

All these measures (or metrics) are based on enumeration of links or shortest paths. In details, let us define a path from 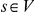 to 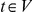, with 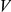 the set of nodes, as an alternating sequence of nodes and edges, beginning with 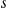 and ending with 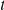, such that each edge connects its preceding with its succeeding vertex. We set the length of a path as to be the sum of the inverse weights of its edges. The idea is that highly correlated miRNAs minimize the distance between nodes or, from another perspective, the closer two nodes are the more they are correlated. Therefore, we compute the distance between two nodes 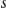 and 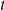, written 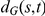, as the minimum length of any path connecting 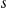 and 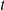 in 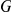. By definition, 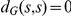 for every 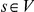.

*Degree* centrality is based on the idea that important nodes are those with the largest number of ties to other nodes in the graph. It is often interpreted in terms of the immediate involvement of nodes in relationships established through the network. Let 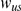 be the weight of the edge, which connects the node 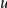 with the node 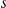, the degree centrality of a node 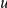 is 
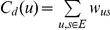
, with 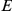 the set of edges, according to which the higher is the degree value, the more important (globally correlated) is the node.

*Betweenness* centrality measures the influence a node has over the indirect correlation between not neighbor nodes. Betweenness, in its basic version, is computed as the fraction of shortest paths between node pairs that pass through the node of interest. Its mathematical expression is 
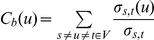
, where 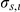 is the number of shortest paths from 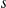 to 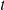, and 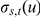 is the number of shortest paths from 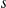 to 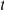 that pass through a vertex 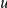. Vertices that occur on many shortest paths between other vertices have higher betweenness, and then higher relevance in indirect correlations.

The *Clustering coefficient* is a measure of degree to which nodes in a graph tend to cluster together, or in other terms, it quantifies nodes because of the extent to which their neighbors are to being a *clique* (complete graph) with it. The clustering coefficient of a node is then given by the proportion of links between the nodes within its neighbourhood divided by the number of links that could possibly exist between them. Succinctly, it is expressed as 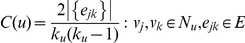, where 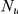 defines the immediately connected neighbours set of 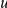 and 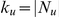. Its ‘weighted’ formulation builds on the weights of the *triangles* centered on the nodes of a network. It is defined by Horvath and Zhang [16] as 
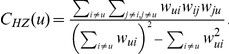
 where 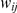 is the weight over the edge 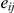, and thus 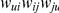 the product of the weights of the edges which form a closed triangle 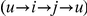 of nodes. Starting from the local definition of the clustering coefficient, we report its averaged formulation by Schank and Wagner [17] for the whole network as the ratio between the sum of the products of the clustering coefficients and a general weight function, and the weight function itself, namely 
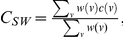
where 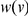 is the general weight function. The weight function is usually chosen among the metrics that better capture the topology of the network under examination. A lower *Average Weighted Clustering Coefficient* measure indicates a more important node in relationship to the network robustness.

Networks have been drawn and analyzed by a custom standalone tool written in C# and built over the library NodeXL 1.0.1.174 [18].

## Results

### Altered miRNA expression in patients with CRC and PC

**Figure 1:**
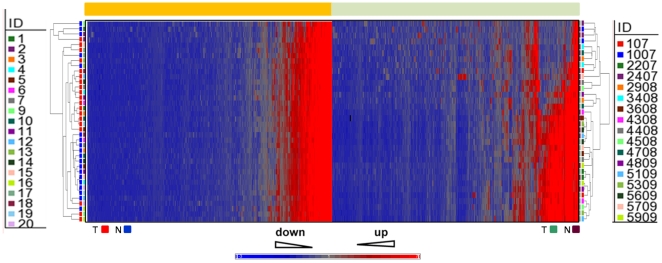
Hierarchical clustering of miRNA expression. miRNA profiles of 36 paired tissue specimens from 19 colorectal cancer (orange box) and 17 pancreatic cancer (green box) patients were clustered. The 36 paired specimens are in rows (coloured bars) and the 866 miRNAs are in columns. **T**, tumor tissue; **N**, adjacent normal tissue.

We compared miRNA profiles of 36 pairs of solid tumors and adjacent nontumorous tissues in the training cohorts (19 CRC and 17 PC) by means of microRNA microarrays. Human miRNAs (*has-miR-*) and tissues were grouped by a hierarchical clustering analysis (Figure 1). Each plotted probe was color-coded to equate the level of expression of the miRNA relative to its median expression level across the entire tissue samples set (blue, low; red, high) (Figure 1).

### Differentially expressed miRNA in CRC and PC

In order to identify miRNAs differentially expressed in paired normal and tumor tissues, two approaches were followed: a classical paired t-test controlling for false discovery rate (fdr) and a RF analysis. By the first analysis, 42 miRNAs were differentially expressed in CRC (Table 1). Twenty-five of them were overexpressed, with *hsa-miR1246* showing the highest fold-change value (12.0-fold). In PC, 128 miRNAs were differentially expressed (see Table S2). In particular, of 34 miRNAs with a p<0.01, 30 showed higher expression levels in tumor tissue, with *hsa-miR-23a* displaying the highest fold-change value (9.3-fold), whereas 4 more miRNAs were down-regulated (Table 2).

**Table 1:** MicroRNAs selected by t-test in CRC.

MiRNA	p-value	Ratio§	Fold-Change
hsa-miR-1246	6.90E-13	12.019	12.0189
hsa-miR-378	4.16E-09	0.320	−3.12036
hsa-miR-28-3p	1.48E-07	0.361	−2.7707
hsa-miR-139-5p	5.74E-07	0.154	−6.50147
hsa-miR-21*	1.10E-06	3.363	3.36277
hsa-miR-140-3p	2.04E-06	0.475	−2.10544
hsa-miR-21	1.38E-05	3.440	3.43984
hsa-miR-145	1.48E-05	0.449	−2.22598
hsa-miR-30c	2.02E-05	0.599	−1.67026
hsa-miR-106a	2.38E-05	1.622	1.62216
hsa-miR-17	2.42E-05	1.733	1.73316
hsa-miR-30d	2.53E-05	0.717	−1.39392
hsa-miR-486-5p	2.58E-05	0.224	−4.46696
hsa-miR-503	2.84E-05	3.357	3.35674
hsa-miR-1290t	5.52E-05	2.296	2.29618
hsa-miR-31	5.58E-05	11.485	11.4851
hsa-miR-342-3p	5.89E-05	0.591	−1.69151
hsa-miR-1826	7.99E-05	2.478	2.47797
hsa-miR-182	8.38E-05	3.694	3.69436
hsa-miR-28-5p	0.000122245	0.487	−2.05143
hsa-miR-429	0.000163538	2.012	2.0116
hsa-miR-10b	0.000179917	0.358	−2.79048
hsa-miR-196b	0.000254369	2.119	2.11937
hsa-miR-1280	0.000286619	2.145	2.14475
hsa-miR-18a	0.000294119	3.826	3.82618
hsa-miR-720	0.000300471	4.459	4.45944
hsa-miR-708	0.000311099	3.459	3.45926
hsa-miR-224	0.000329656	2.320	2.3199
hsa-miR-143	0.000408223	0.512	−1.95475
hsa-miR-183	0.000474118	3.064	3.06428
hsa-miR-138	0.000528794	0.302	−3.31458
hsa-miR-422a	0.000533316	0.306	−3.26738
hsa-miR-99 a	0.000577088	0.340	−2.94193
hsa-miR-18b	0.000690552	2.521	2.52109
hsa-miR-195	0.000701403	0.436	−2.2921
hsa-miR-149	0.000758911	0.494	−2.02318
hsa-miR-92a-1*	0.000766587	1.905	1.90481
hsa-miR-339-5p	0.000883788	3.056	3.0557
hsa-miR-19a	0.0009656	2.268	2.26773
hsa-miR-19b	0.00103109	1.914	1.91352
hsa-miR-130b	0.00104793	2.053	2.05348
hsa-miR-92a	0.00105709	2.322	2.32229

Forty-two miRs deregulated in tumoral compared to matched normal tissue and selected by p<0.001. Twenty-five overexpressed miRs in cancer are indicated by positive fold-change; seventeen down-regulated miRs are indicated by negative fold-change.

§ : Tumour vs Normal tissue.

**Table 2:** MicroRNAs selected by t-test in PC.

MiRNA	p-value	Ratio§	Fold-Change
hsa-miR-23a	0.000215491	9.37178	9.37178
hsa-miR-1254	0.000567431	0.578601	−1.72831
hsa-miR-103	0.00116796	5.22503	5.22503
hsa-miR-107	0.0015613	6.83561	6.83561
hsa-miR-1207-5p	0.00156319	2.27566	2.27566
hsa-miR-125a-5p	0.00173021	6.55499	6.55499
hsa-miR-221	0.00175528	3.74587	3.74587
hsa-miR-140-5p	0.00181141	1.47229	1.47229
hsa-miR-143	0.00201799	6.38038	6.38038
hsa-let-7d	0.00221191	5.26385	5.26385
hsa-miR-146°	0.00221576	3.06742	3.06742
hsa-miR-145	0.00236652	5.76811	5.76811
hsa-let-7e	0.00265301	7.05982	7.05982
hsa-miR-199b-3p	0.00275494	4.79528	4.79528
hsa-miR-138-1*	0.00294212	1.71977	1.71977
hsa-miR-92b	0.00306909	1.75529	1.75529
hsa-miR-199a-3p	0.00333868	4.63266	4.63266
hsa-miR-29b-1*	0.00372484	1.34857	1.34857
hsa-miR-92°	0.00391816	5.19232	5.19232
hsa-let-7f-1*	0.00402756	0.669364	−1.49396
hsa-miR-559	0.00419118	0.499458	−2.00217
hsa-miR-181°	0.00473953	4.4176	4.4176
hsa-miR-1246	0.00499233	2.37905	2.37905
hsa-miR-31	0.00534964	4.1793	4.1793
hsa-let-7°	0.0057542	5.60946	5.60946
hsa-miR-331-3p	0.00587375	1.91755	1.91755
hsa-miR-155	0.00659337	3.93778	3.93778
hsa-miR-1274a	0.0072345	0.74278	−1.34629
hsa-miR-26a	0.0075564	5.1572	5.1572
hsa-miR-17	0.00851766	3.07585	3.07585
hsa-miR-23b	0.00916704	5.8671	5.8671
hsa-miR-24	0.00928757	4.58821	4.58821
hsa-miR-939	0.0093498	1.52377	1.52377
hsa-miR-500*	0.00996664	2.34195	2.34195

Thirty-four miRs deregulated in tumoral compared to matched normal tissue and selected by p<0.01. Thirty overexpressed miRNAs in cancer are indicated by positive fold-change; four down-regulated miRNAs are indicated by negative fold-change.

§ : Tumour vs Normal tissue.

To ascertain the eventual existence of orthologues in other species, and to use them as internal methodological control, the human miRNAs found altered in the two solid tumors were checked in 71 other organisms present in the array: all de-regulated orthologues of human miRNAs (from now on, *has-miR-* will be referred to as *miR-*) showed a significant differential expression with similar fold-change in these organisms (Table S3).

**Figure 2:**
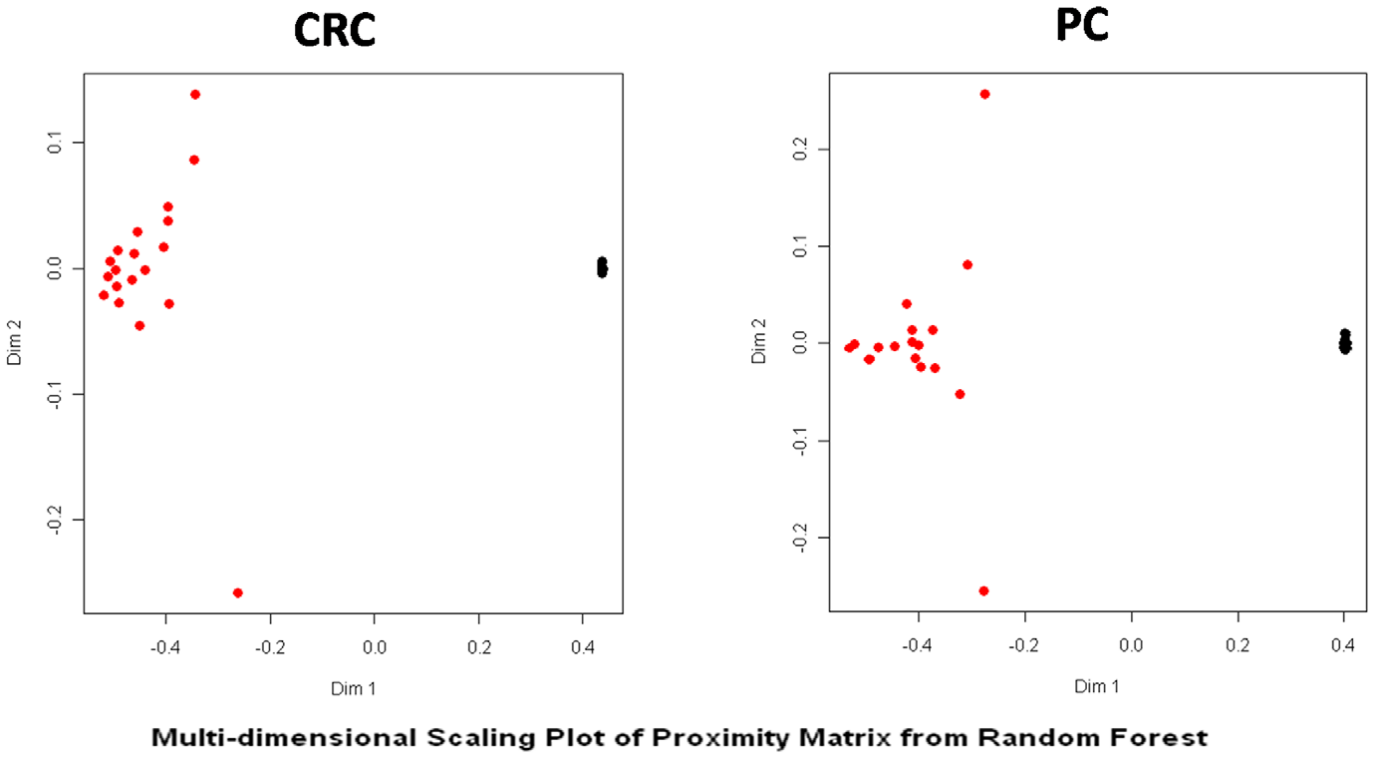
Multidimensional scaling plot. Proximity matrices from random forest analysis, where *x-* and *y-* axes are the multidimensional scaling coordinates. Subjects with similar miRNA expressions are represented by points close one to the other, whereas subjects with dissimilar miRNA expressions are represented by separated points. Red = tumor tissue, Black = adjacent normal tissue.

**Figure 3:**
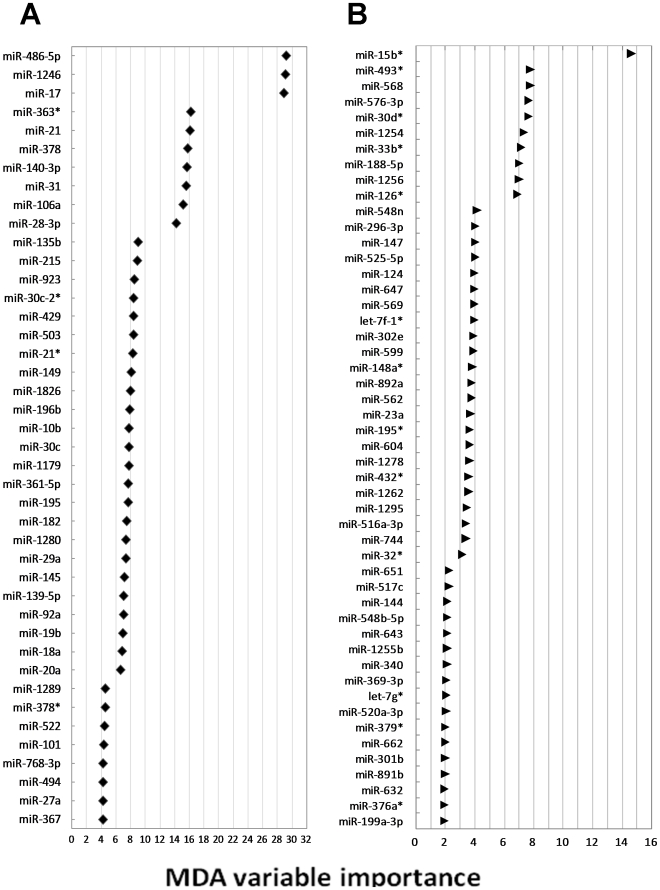
Variable Importance table. Mean Decrease in Accuracy (MDA) as a measure of miRNA importance in classifying tumor tissues from normal ones estimated by random forest analysis. The first 42 most important miRNAs in colorectal cancer (panel **A**) and 50 miRNAs in pancreatic cancer (panel **B**) are shown.

To check the accuracy of the RF, classification performances in discriminating tumor from normal tissue were reported in multidimensional scaling of the estimated proximity matrix plots (Figure 2) displaying similarities or dissimilarities in data. For each tumor type, the ranks of the most important miRNAs, according to MDA, were derived (Figure 3, panels A and B).

### Comparison of miRNA signature detection methods

**Figure 4:**
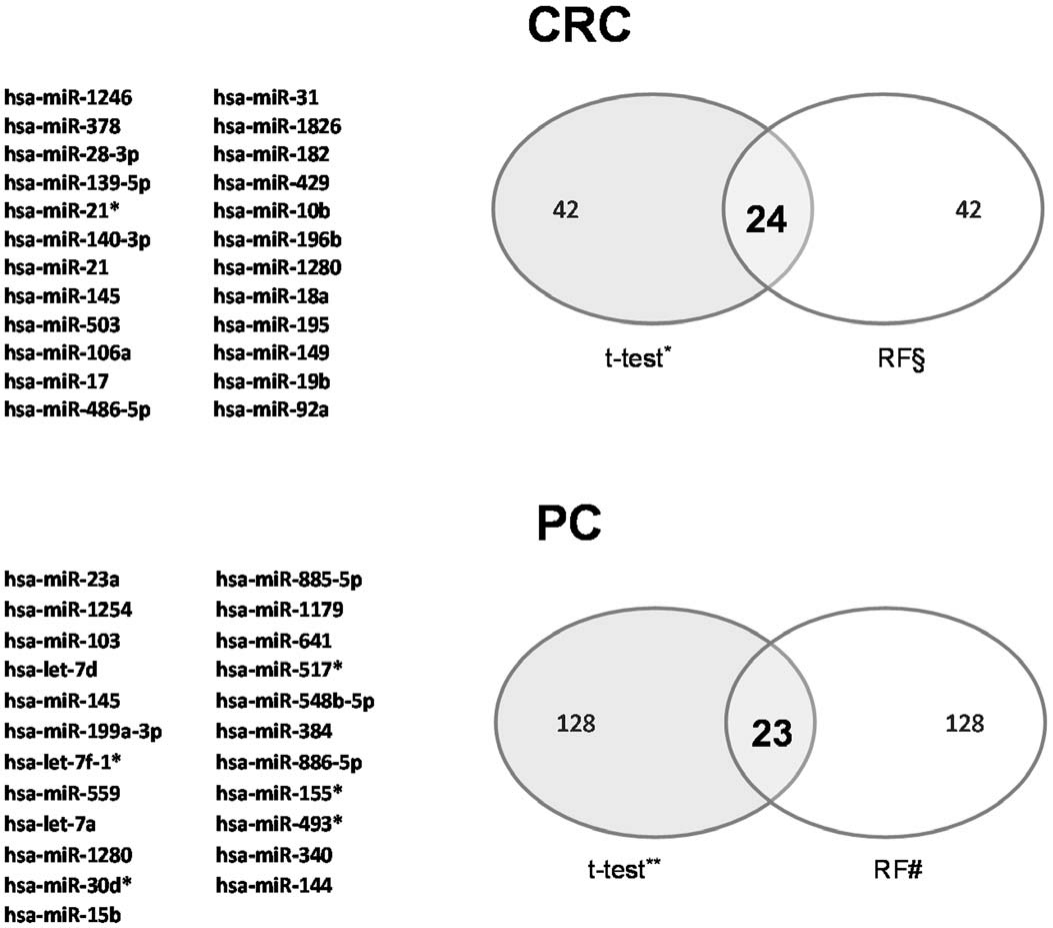
Merged results. For both tissues a significant overlap among detected miRNAs was found by merging t-test and RF results. A group of 24 miRNAs, whose expression was significantly altered in colorectal tumors, were obtained intersecting the list of first 42 miRNAs with best p-values, from t-test analysis, with those at highest MDA, from RF analysis. In the same fashion, 23 miRNAs with altered expression were selected in pancreatic tumors. * p<0.001; § RF>4.3; **p<0.05; # RF>0.94.

When the results with the t-test and RF were merged, a significant overlap among miRNAs detected in either CRC and PC tissues was found. By intersecting the miRNAs with p<0.001 at t-test analysis (Table 1) with those with the highest MDA at RF analysis (Figure 3A), the expression of 24 miRNAs was significantly altered in CRC (Figure 4). By a similar analytical approach (Table S2, and Figure 3B), 23 miRNAs were significantly altered in PC (Figure 4).

As shown in Figure 4, miRNA expression was heavily tissue specific; indeed, most miRNAs with altered expression in CRC were not differentiallly expressed in pancreatic tumoral tissue. The altered expression of only two miRNA was shared by CRC and PC: *miR-145* appeared 2.2-fold down-regulated (p<0.001) in CRC, and 5.7-fold up-regulated (p<0.01) in PC, while *miR-1280* was 2.1-fold up-regulated (p<0.001) and 2.3-fold down-regulated (p<0.05) in the former and latter cancers, respectively (see Table 1, Table 2 and Table S2).

### miRNA correlations and cancer-miR network

Next we tried to verify the inter-relationship of previously selected miRNAs expressed in either CRC and PC tissues by means of the Spearman correlation coefficient method.

**Figure 5:**
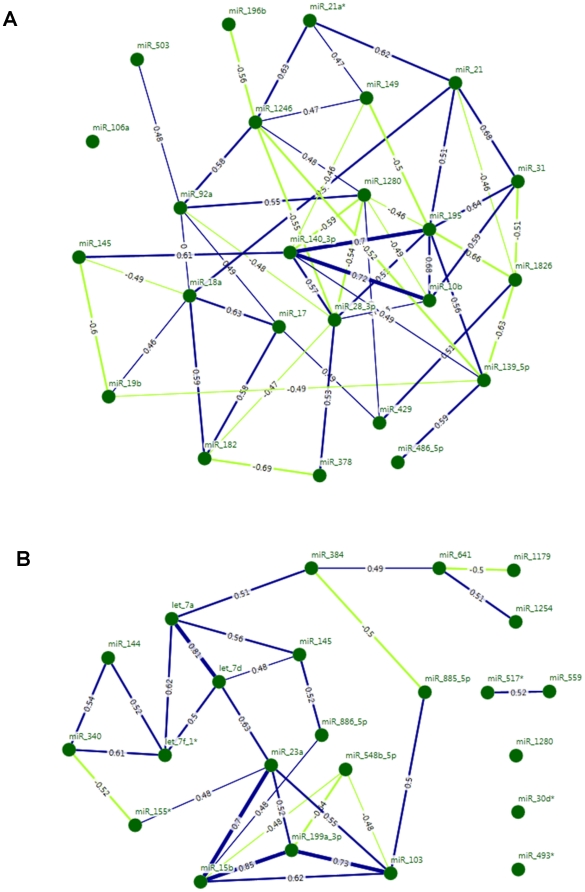
CRC and PC network. Green points depict the nodes corresponding to 24 miRNAs of CRC network and 23 miRNAs of PC network. The edges characterize the significant correlations, whose thickness reflects Spearman Correlation Coefficients (blue and green edges are used for positive and negative correlations respectively). In CRC network, as shown in panel **A**, m*iR-195, miR-28-3p, miR-1280, miR-18a* and *miR-1246* exhibit the highest *weighted* degree rank. No correlation is found for *miR-106a*. *MiR-1246* has also the highest *betweenness* rank. In PC network, as shown in panel **B**, *miR-103, miR-23a* and *miR-15b* have the highest *degrees* and are all linked to *miR-199a-3p* as well as to each other forming a four nodes *clique*. In addition, the removal of these nodes causes the deepest drop of the average clustering coefficient rank. *MiR-384* exhibits the highest betweenness centrality measure.

For CRC, different *degrees* of correlation were found for the 24 miRNAs exhibiting altered expression: *miR-106a* was not correlated at all, while *miR-195* exhibited the largest number of ties and was taken as a root node with 9 edges (*degree* 15.86); 8 links were detected for *miR-28-3p* (*degree* 15.66), and 7 links for *miR-1280* (*degree* 13.79), *miR-1246* (*degree* 13.05), and *miR-140-3p* (*degree* 12.12) (Figure 5, panel A). The latter 3 nodes were also interconnected with *miR-28-3p*, which showed a high betweenness value. For this last measure, the highest value was observed for *miR-1246* node on which many shortest paths between other vertices occur (Table S4). Another important node was represented by *miR-18a* with 6 relationships, 3 of them established with several miRNAs belonging to the *miRNA 17-92a* cluster. When we measured the degree of nodal clustering (c*lustering coefficient*), the *miR-378, miR-10b* and *miR-31* appeared to be a *cliqu*e with vertice in *miR-28-3p*. To verify the robustness of the network, we calculated the *average weighted clustering coefficient*, and evaluated the contribution of a miRNA to the overall compactness of the network. The lowest scores were observed for *miR-1280, miR-195* and *miR-140-3p* which were the vertices of complete graph (Figures 5, panel A and Table S4).

Twenty out of 23 miRNAs with a significant deregulation in PC tissues were linked to others vertices of the network by a number of edges varying from one to five (*degree*: from 8.88 to 1.92); for only 3 miRNAs (i.e. miR-30d*, miR-1280 and miR-493*) no significant correlation was observed (*degree*: 0), (Figure 5, panel B). In particular, the maximum number of ties was found for *miR-103* (*degree*: 8.88), *miR-23a* (*degree*: 8.84) and *miR-15b* (*degree*: 8.38). These 3 main nodes were all linked to *miR-199a-3p* (degree: 6.03) and these four miRNA appeared inter-crossed to constitute a four-node clique. The highest betweenness centrality measure was observed for *miR-384* (*betweenness*: 0.31), found to be critically positioned in between (thus, responsible of many) medium-range correlations. We verified the essentiality of the four-node *clique* by measuring the overall robustness of the network (in terms of paths redundancy) after removing it. We computed the *average weighted clustering coefficient* for the whole network by removing in turn a node. In particular, after excluding the nodes of the *clique* (*miR-103, miR-15b, miR-199a-3p* and *miR-23a*) the lowest ranks, namely 5.78, 5.85, 6.05 and 6.14 were obtained, respectively. This finding pinpoints to these nodes as functionally relevant and as considerable constituent of the cohesiveness of the network (Figure 5, panel B and in Table S4).

### Validation qPCR

Among significantly deregulated miRNAs in the microarray study, 18 were selected for further validation by quantitative real-time PCR (qPCR). Selection of these miRNAs was based on two criteria: the outcome of the previously described intersection analysis, and/or their involvement in colon or pancreas tumorigenesis (according to literature data). Quantitative PCR was applied to analyse altered expression of the selected miRNAs, as well as that of the RNAU6 control, in the tumor and normal tissues taken from a new validation cohorts of 14 patients with CRC and 21 PC patients.

Compared to normal tissue with an expression profile normalized to 1, in tumor samples of the 14 CRC patients we observed a significant up-regulation for 3 miRNAs (*miR-31, miR-21* and *miR-708*), and under expression in 7 others (*miR-145, miR-139-5p, miR-486-5p, miR-378, miR-140-3p, miR-143* and *miR-30c*) (Table 3). The remaining 8 miRNAs (miR-151-5p, miR-155*, miR-17, miR-199a-5p, miR-23a, miR-30a-5p, miR-455-3p, and miR-let-7i) did not exhibit significantly altered expression.

**Table 3:** Differentially regulated miRs in CRC and in PC vs. matched respective normal tissues.

Variable	mean fold-change (Q1-Q3)		P value	Cancer type
*miR-31*	28.01	(5.75–68.59)	<0.0001	Colon
	12.29	(3.03–29.14)	<0.0001	Pancreas
*miR-21*	2.88	(1.20–4.70)	<0.05	Colon
	0.80	(0.19–2.94)	ns	Pancreas
*miR-708*	1.93	(0.65–3.53)	<0.05	Colon
	3.62	(1.16–11.24)	<0.05	Pancreas
*miR-143*	0.30	(0.18–1.18)	<0.05	Colon
	3.52	(0.95–9.55)	<0.0001	Pancreas
*miR-30c*	0.36	(0.14–0.77)	<0.05	Colon
	1.36	(0.38–3.44)	ns	Pancreas
*miR-151-5p*	0.77	(0.41–1.58)	ns	Colon
	1.12	(0.76–2.68)	<0.05	Pancreas
miR-155*	1.34	(0.46–3.91)	ns	Colon
	4.07	(1.15–38.17)	<0.05	Pancreas
*miR-199a-5p*	0.65	(0.30–1.34)	ns	Colon
	2.14	(0.34–15.13)	<0.05	Pancreas
*miR-23a*	1.44	(0.88–2.26)	ns	Colon
	2.91	(0.81–13.63)	<0.05	Pancreas
*miR-455-3p*	1.03	(0.65–2.47)	ns	Colon
	1.62	(0.69–4.95)	ns	Pancreas
*miR-30a-5p*	0.71	(0.29–1.34)	ns	Colon
	1.24	(0.07–2.26)	ns	Pancreas
*Let-7i*	1.07	(1.03–1.86)	ns	Colon
	1.77	(0.68–7.07)	<0.05	Pancreas
*miR-145*	0.20	(0.08–0.26)	0.0001	Colon
	2.72	(0.64–6.89)	<0.05	Pancreas
miR-486-5p*	0.43	(0.25–0.83)	<0.0001	Colon
*miR-378*	0.23	(0.11–0.60)	<0.05	Colon
*miR-140-3p*	0.46	(0.14–0.96)	<0.05	Colon
*miR-139-5p*	0.10	(0.04–0.14)	<0.05	Colon
*miR-17*	0.97	(0.36–2.13)	ns	Colon

In the cohort of 21 PC patients, 13 out of 18 miRNAs were analyzed by qPCR in tumoral tissue compared to normal samples. Down-expression was observed only for *miR-21*, whereas over-expression was observed in 12 miRNA (miR-143, miR-145, miR-151-5p, miR-155*, miR-199a-5p, miR-23a, miR-30a, miR,30c, miR-21, miR-455-3p, miR-708 and *miR-let-7i*) and only two were not deregulated in a statistically significant way (*miR-30a and miR-30c*) (Table 3).

## Discussion

MiRNAs that are differentially expressed in various cancers may participate in common altered regulatory pathways [19]. Thus, the knowledge of their interacting network can provide a perspective of altered miRNAs and their pattern of deregulation (i.e., up- or down-regulation). In this study, we profiled miRNAs in two different solid cancer tissues, the CRC and PC, to test whether any plausible “signatures” was detectable. To this purpose, we initially looked for miRNAs differentially expressed in tumors and normal tissue of the two different lineages, and subsequently identified “tissue-specific” miRNAs by using a novel statistical approach, i.e. the RF classifier. For each cancer tissue we were able to generate a list of the most specific and significantly deregulated miRNAs. Interestingly, in the two lists only two miRNAs recurred but with opposite patterns of expression: *miR-145* and *miR-1280*. However, while different patterns of the expression of *miR-145* are recognized [20], nothing is known about *miR-1280* and its behavior in solid tumor.

A comprehensive analysis of interactive networks of these miRNAs showed that their deregulation patterns are tissue specific, as dissimilar connections (i.e., different correlation patterns) were observed between them in the two lineages. Moreover, we determined the most critical miRNAs and verified that they were different in CRC and in PC networks.

To understand the regulatory mechanisms of the miRNAs linked with significant connections in both networks, we conducted a three-step *in-silico* analysis. First, we ranked nodes by means of well-known centrality indices: weighted *degree* and *betweenness*. While the former index provided insights of the direct effect exerted by each node to its neighborhood (in our case, their correlation), the latter identified nodes essential for indirect correlations, namely nodes that lie in paths of correlation and that somehow contributes to long range correlations. Consequently, we ranked nodes with the *clustering coefficient* index, in order to measure their degree of cohesiveness with the immediate neighbors. The aim was to quantify the robustness of the networks, by looking for the nodes that would otherwise disrupt the networks architecture when not taken into account. To this purpose, we computed the cluster coefficient for the whole networks by cyclically removing one node with corresponding edges, and by employing the *weighted degree* as weight function. Generally, networks lacking nodes essential to the overall robustness exhibited lower scores.

As last step, we validated our *in-silico* estimations by manually inspecting target genes and their signaling pathways. With these approaches, we observed that the majority of miRNAs with similar deregulation patterns were associated with common target genes and/or regulatory pathways (see Table S5). In particular, in CRC three principal nodes were found: *miR-195, miR-18a*, and *miR-1246*. The first node had a high relationship with miRNAs involved in MAPK signaling pathway, as shown *in-silico* analysis by using DIANA-mirPath (http://diana.cslab.ece.ntua.gr/pathways/index_multiple.php), except *miR-31 and miR-28-3p*, which belong to the EGF pathway. The second node, the *miR-18a*, included several miRNAs (*miR-17, miR-19b, miR-92a*) belonging to the same cluster: the *miRNA 17-92a*. This cluster plays an important role in carcinogenesis and embryogenesis by regulating the proliferation process through *E2F1* gene under-expression [21]. The other three correlations (*miR-145, miR-21* and *miR-182*) were found associated with IGF1R [22], EGFR [23] and AKT [24] signaling pathway. The last relationship has *miR-1246* as principal node. Recently, Zhang et al. [25] have shown that *miR-1246* is a new target of p53 family members. TP53 induces the expression of *miR-1246* which, in turn, reduces the level of DYRK1A, a Down syndrome-associated protein kinase. Overexpression of *miR-1246* reduces DYRK1A levels and decreases the induction of apoptosis [25].

Focusing on the principal nodes detected in PC network, *miR-103* was associated with alteration of TGF-β signaling pathway, and *miR-23a* with KRAS-mediated signaling pathway, while for *miR-15b* a number of biological relevant associations with cellular signaling pathways were observed [26]. On the other hand, the commonly correlated node *miR-199a-3p*, which form a complete graph with the three main vertices of the network, was associated with MAPK signaling pathway. It is to note that both TFG-β and KRAS pathways belong to the “core signaling pathways” and processes reported to be altered in many PC [26]. In addition, considering that a lower *Average Weighted Clustering Coefficient* measure indicates a more important node in relationship to the network robustness, the low value obtained for *miR-103* underlines the central role for TGF-β signaling in PC, in agreement with previous observations [27]. Moreover, when we performed *in-silico* analysis of these four miRNAs inter-crossed to form a *clique* by using DIANA-mirPath, (http://diana.cslab.ece.ntua.gr/pathways/index_multiple.php), a previously undescribed link with Focal Adhesion pathway was put in evidence.

The approaches used in this study allowed us to connect deregulation of miRNA expression to oncogenic pathways and identify links undescribed up until now between altered specific miRNA expression and pathways involved in organ-specific cancer: even though the oncogenic pathways identified in both cancers were similar, the miRNAs linked to them were specific for CRC or PC highlighting the organ-specificity of miRNA networks.

In conclusion, our observations highlight network connections between miRNAs in CRC and PC, and suggest that the regulatory miRNAs, as network nodes, might have combinatorial effects on driving specific cellular regulatory pathways involved in cancer development and progression. Data obtained in our study encourage to further investigate details of miRNA involvement in signaling pathways deregulated in CRC and PC, and to understand whether correlations between specific miRNAs might be in turn accompanied by connections between the respective influenced signaling pathways.

## Supporting Information

Table S1 **Clinical and pathological features of colorectal cancer and pancreatic cancer patients.**
(DOC)

Table S2 **miRNAs differentially expressed in pancreatic cancer (PC) by t-test analysis.**
(XLS)

Table S3 **Altered human miRNAs in solid tumors in other organisms.**
(XLS)

Table S4 **Centrality Measures For CRC-specific and PC-specific Network.**
(XLS)

Table S5 **Signaling Pathways of miRNA target in colorectal and pancreatic cancer.**
(DOC)
