# Supplementary material for: Mirna Expression Profiles Identify Drivers in Colorectal and Pancreatic Cancers
Source: PLoS One. 2012 Mar 30;7(3):e33663. doi: 10.1371/journal.pone.0033663 (PMC3316496; doi:10.1371/journal.pone.0033663)
Supplement: Supplementary file 1 [file pone.0033663.s001.doc]

Clinical and pathological features of colorectal cancer (CRC) and pancreatic cancer (PC) patients

**Clinical Data**	**CRC** training cohorts n= 19 (%)	**CRC** validation cohorts n= 14 (%)
**Age (yrs):**		
Mean (±SD)	67 ± 2	62 ± 14
**Sex:**		
Male/Femal	12/7	11/3
**Tumour location:**		
Ascending colon	4 (21%)	7 (50%)
Descending colon	3 (16%)	7 (50%)
Rectum-Sigmoid	12 (63%)	0
**Histopathologic Type:**		
Adenocarcinoma	15 (79%)	10 (71%)
Mucinous	4 (21%)	4 (29%)
**Histopathologic**		
G1/G2	17 (89%)	11 (79%)
G3/G4	2 (11%)	3 (21%)
**DUKE'S:**		
A	3 (16%)	0
B	9 (47%)	11 (79%)
C	7 (37%)	3 (21%)

**Clinical Data**	**PC** training cohorts n= 17 (%)	**PC** validation cohorts n= 21 (%)
**Age (yrs):**		
Mean (±SD)	64 ± 15	65.7 ± 11.45
**Sex:**		
Male/Femal	11/6	16/5
**Histopathologic Type:**		
PDAC	9 (53%)	13 (61.9%)
PVAC	5 (29%)	6 (28.6%)
IP-CCA	2 (12%)	-
PNET	1 (6%)	2 (9.5%)
**Histopathologic Grade**		
G0	1 (6%)	2 (9.5%)
G1	2 (12%)	4 (19%)
G1-G2	1 (6%)	1 (4.8%)
G2	7 (41%)	10 (47.6%)
G2-G3	5 (29%)	3 (14.3%)
G3	1 (6%)	1 (4.8%)
**Tumour stage**		
0	1 (6%)	2 (9.5%)
II	1 (6%)	-
IIA	2 (12%)	2 (9.5%)
IIB	7 (41%)	11 (52.4%)
III	4 (23%)	6 (28.6%)
IV	2 (12%)	-
