# Supplementary material for: Mirna Expression Profiles Identify Drivers in Colorectal and Pancreatic Cancers
Source: PLoS One. 2012 Mar 30;7(3):e33663. doi: 10.1371/journal.pone.0033663 (PMC3316496; doi:10.1371/journal.pone.0033663)
Supplement: Supplementary file 2 [file pone.0033663.s005.doc]

Signaling Pathways of miRNA target in colorectal and pancreatic cancer

CRC

miRNA		***Signaling Pathway***
Principal Nodes	***Relationships***	
miR-195	*miR-10b*	*MAPK*
	miR-140-3p	*MAPK*
	miR-28-3p	*MAPK*
	miR-149	*MAPK*
	miR-139-5p	*MAPK*
	miR-31	*EGF*
	miR-21	*EGF*
miR-18a	*miR-17*	*E2F1*
	miR-19b	*E2F1*
	miR-92a	*E2F1*
	miR-145	*IGF1R*
	miR-182	*AKT*
	miR-21	*EGFR*
miR-1246		*TP53*
	miR-196b	
	*miR-21a**	
	miR-149	
	miR-1280	
	miR-139-5p	
	miR-28-3p	
	miR-92a	

PC

miRNA		***Signaling Pathway***
Principal Nodes	***Relationships***	
miR-103		*TGF-B*
miR-23a		*KRAS*
miR-15b		*BCL2*
miR-199a-3p		*MAPK*
miR-199a-3p	*miR-103*	*FOCAL ADHESION*
	miR-23a	
	miR-15b	
